# The *SPRED1* Variants Repository for Legius Syndrome

**DOI:** 10.1534/g3.111.000687

**Published:** 2011-11-01

**Authors:** Kelli Sumner, David K. Crockett, Talia Muram, Kalyan Mallempati, Hunter Best, Rong Mao

**Affiliations:** * ARUP Institute for Clinical and Experimental Pathology, Salt Lake City, Utah 84108; † Department of Pathology, University of Utah School of Medicine, Salt Lake City, Utah 84112

**Keywords:** Legius syndrome, *SPRED1*, *NF1*, database

## Abstract

Legius syndrome (LS) is an autosomal dominant disorder caused by germline loss-of-function mutations in the sprouty-related, EVH1 domain containing 1 (*SPRED1*) gene. The phenotype of LS is multiple café au lait macules (CALM) with other commonly reported manifestations, including intertriginous freckling, lipomas, macrocephaly, and learning disabilities including ADHD and developmental delays. Since the earliest signs of LS and neurofibromatosis type 1 (NF1) syndrome are pigmentary findings, the two are indistinguishable and individuals with LS may meet the National Institutes of Health diagnostic criteria for NF1 syndrome. However, individuals are not known to have an increased risk for developing tumors (compared with NF1 patients). It is therefore important to fully characterize the phenotype differences between NF1 and LS because the prognoses of these two disorders differ greatly. We have developed a mutation database that characterizes the known variants in the *SPRED1* gene in an effort to facilitate this process for testing and interpreting results. This database is free to the public and will be updated quarterly.

Legius syndrome (LS; MIM #611431) is an inherited disease caused by autosomal dominant loss-of-function (LOF) mutations in the sprouty-related, EVH1 domain containing 1 (*SPRED1*) gene (MIM #609291) (Brems *et al.* 2007). LS, also known as neurofibromatosis type 1 (NF1)-like syndrome, is a disorder with a pigmentary phenotype overlapping with NF1 syndrome. Individuals with LS may meet the diagnostic criteria set by the National Institutes of Health (NIH) for NF1 syndrome (Brems, *et al.*, 2007; Pasmant, *et al.*, 2009; Stevenson, *et al.* 2010), except people with LS have not been shown to develop tumors as found in NF1(Muram-Zborovski *et al.* 2010). Additional manifestations associated with LS have also been reported in individuals with NF1 (*i.e.*, macrocephaly, lipomas, learning difficulties, Noonan syndrome-like facial features, attention deficit and hyperactivity disorder (ADHD), and pectus excavatum (Brems *et al.* 2007; Friedman 1998; Muram-Zborovski *et al.* 2010; Pasmant *et al.* 2009). For a clinical diagnosis of NF1, an individual must meet two or more of the following criteria: six or more café-au-lait macules (CALM); two or more neurofibromas or one plexiform neurofibroma; freckling in the axillary or inguinal regions; optic glioma; two or more Lisch nodules; distinctive osseous lesion; and/or a first-degree relative with the diagnosis of NF1. LS patients may meet the NIH diagnostic criteria for NF1 based on pigmentary findings of café-au-lait macules and distinctive freckling pattern alone. It is therefore important to fully characterize the phenotype differences between LS and NF1 because the prognoses for these two disorders differ significantly. This is especially true in children, as definitive features of NF1 are acquired with age, and the only manifestation may be CALM and axillary/inguinal freckling, making it difficult to differentiate between LS and NF1 without genetic testing.

The *SPRED1* gene, located on chromosomal region 15q3.2, contains seven coding exons and produces a protein that is a negative regulator of the RAS-MAPK signaling pathway. It is believed that SPRED1 interacts directly with RAS-RAF signaling and that most germline mutations in the *SPRED1* gene result in a truncated protein that is incapable of properly interacting with the RAS pathway. However, pathogenic missense mutations, as well as large deletions encompassing part of or the entire *SPRED1* gene, have been described, suggesting that many different types of mutations are capable of resulting in protein products unable to properly regulate the RAS-MAPK pathway. These mutations can produce a LS phenotype and may complicate the interpretation of genetic testing results. As such, clinical and research laboratories that offer *SPRED1* gene analysis often encounter rare variations and must rely on the published literature or online resources to determine the clinical relevance of the identified variant. We have developed a database devoted to LS-related mutations in the *SPRED1* gene to facilitate this process.

We have recently reported gene variant archives for galactosemia (*GALT*), multiple endocrine neoplasia type 2 (*RET*), juvenile polyposis syndrome (*SMAD4*), and X-linked Alport syndrome (*COL4A5*). These clinically curated archives uniquely display genotype and associated phenotype information. Here, we introduce a new disease database for LS syndrome and *SPRED1* gene variants. This database was developed to serve as a reference for all sequence variations currently reported in the *SPRED1* gene as well as to provide as much phenotype information as possible (Calderon *et al.* 2007; Crockett *et al.* 2010; Margraf *et al.* 2009; Wooderchak *et al.* 2010). To date, 78 variants have been described in the *SPRED1* gene, of which 49 have been classified as disease causing or pathogenic, 1 as suspected pathogenic, 7 as suspected benign, 8 as benign polymorphisms, and 13 with uncertain significance (Brems *et al.* 2007; Denayer *et al.* 2011; Messiaen *et al.* 2009; Muram-Zborovski *et al.* 2010; Pasmant *et al.* 2009; Spencer *et al.* 2011; Spurlock *et al.* 2009).

## *SPRED1* variant database objectives

The aim of the *SPRED1* variant database is to record all known variants in the *SPRED1* gene and their associated phenotype. This database is freely available to the public, and users are encouraged to submit newly discovered variants to the database curator using the “Database Submission” webpage. This form also can be used to update clinical information or to clarify the classification or phenotype for existing sequence variants. The submission form requests information on the sequence variation, clinical consequences, publications, and the submitter’s contact information. Those submitting new variants to the database are expected to follow their own Institutional Review Board protocols or consenting processes, as their host institutions deem necessary. To maintain the accuracy and utility of the *SPRED1* database, content will be updated quarterly using new sequence variation information from literature reports, database submissions and updates, and routine clinical testing performed at ARUP Laboratories. The original start date and date of the latest update is displayed on the *SPRED1* “Home” webpage.

## Methods

### Data sources and limitations

The *SPRED1* database was constructed using gene sequence variation data published in the scientific literature since the *SPRED1* gene was first identified in patients with a NF1-like phenotype but with no identifiable *NF1* gene mutation in 2007 (Brems *et al.* 2007). To date, 78 sequence variants have been found by searching PubMed (http://www.ncbi.nlm.nih.gov/sites/entrez) and Google (http://google.com/). Search terms included *SPRED1*, *Legius syndrome*, and *NF1-like syndrome*. Occasionally, laboratory-sequencing results that identify novel gene variants are also included in this archive. The database was constructed using the Human Genome Variation Society (HGVS) and Human Genome Organization (HUGO) Mutation Database Initiative recommendations for essential and optional content (http://www.hgvs.org/mdifaq.html). HGVS nomenclature recommendations for description of sequence variants were used to generate DNA and protein changes for this database. Reference sequences for this database were GenBank NC_000015.9, NM_1525940.2, and NP_689807.1; entries were verified for position and name based on these sequences. All sequence variants in the database (including future updates) are named based on their position in relation to the coding regions of the *SPRED1* gene (cDNA) following recommendations listed by the HGVS (http://www.hgvs.org/mutnomen) and described in Den Dunnen and Antonarakis (2000). To comply with sequence variant nomenclature according to the guidelines of the Human Genome Variation Society, the online batch position check tool Mutalyzer 2.0 (http://www.mutalyzer.nl/) was used to confirm genomic position of all *SPRED1* gene variants (Wildeman *et al.* 2008).

### Software

SQL tables (MySQL, Inc.) were organized to represent *SPRED1* mutation status, associated phenotype, literature references, and any known clinical information. PHP coding was performed in-house for dynamic HTML display to render pages and display SQL tables. Graphics were generated using FusionCharts v3 (www.fusioncharts.com). Web pages are hosted on a Mac OS X Apache server. *SPRED1* mutations were added to the database and edited using phpmyadmin (www.phpmyadmin.net).

## Results and Discussion

### *SPRED1* database website

**Figure 1:**
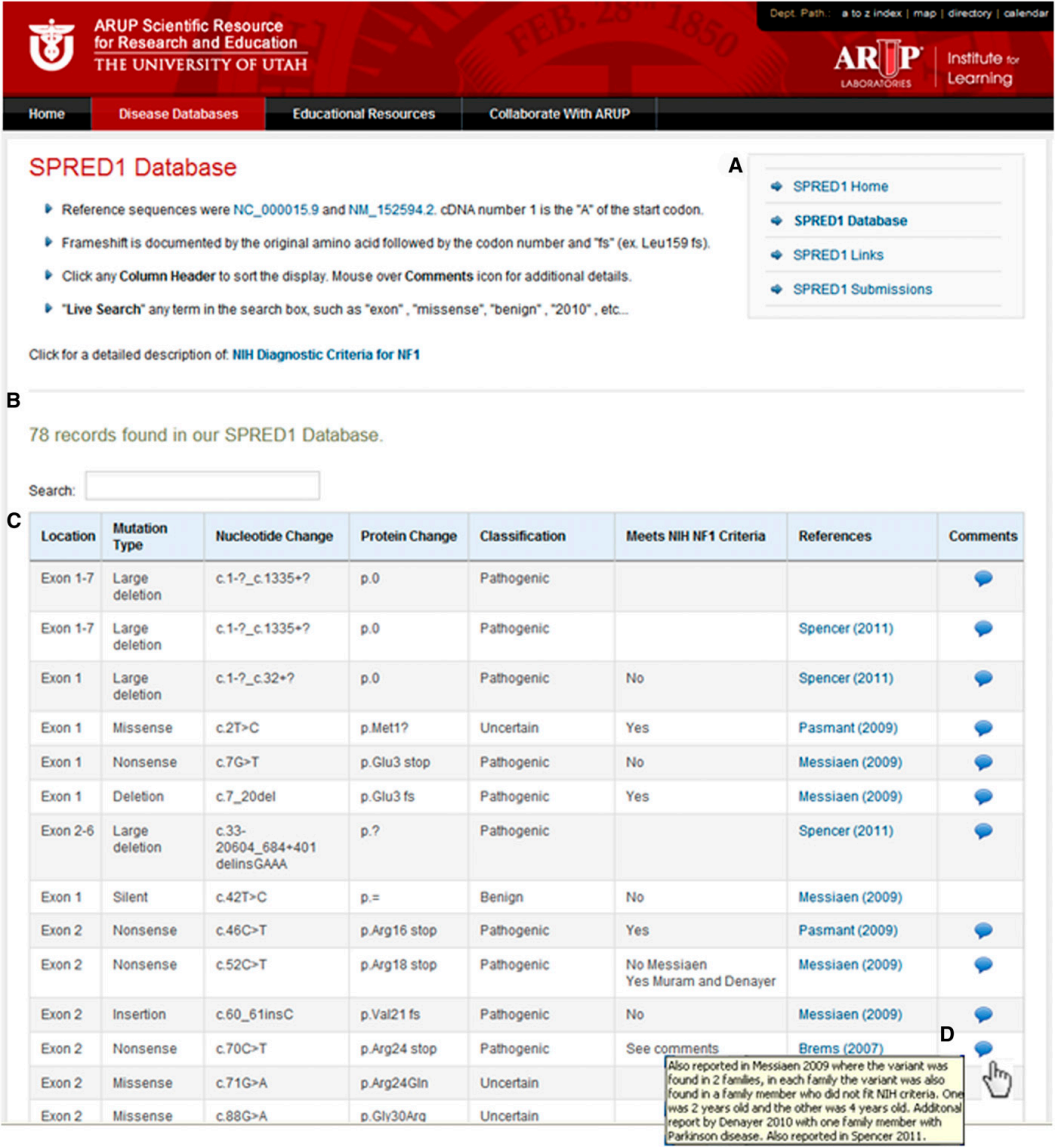
Legius syndrome database display. (A) Quick links for navigation within the website for SPRED1 homepage, database display, links, and submission. (B) Main database display including a “live search” function for easy filtering to gene variants of interest. (C) Clicking any display column heading will sort gene variants in ascending or descending order. (D) Mouse-over function for additional clinical “Comments” column follows the literature reference column.

The *SPRED1* database can be found under Disease Databases on the ARUP Online Scientific Resource webpage http://www.arup.utah.edu/ or accessed directly by using the URL http://www.arup.utah.edu/database/SPRED1/SPRED1_welcome.php. Navigation between the series of webpages can be done using the quick links as shown in Figure 1A. The *SPRED1* “Home” webpage briefly describes Legius syndrome, phenotype, the database goals, reference sequences used, and gene sequencing tests available at ARUP Laboratories. The right frame of the page conveniently lists contact information for the appropriate experts involved in the database development and curation, including medical director, submissions, testing, and website administrator. The “Home” page also provides a graphic link to the *SPRED1* gene topic hosted by the National Library of Medicine’s Genetics Home Reference (http://ghr.nlm.nih.gov/gene/SPRED1).

The *SPRED1* “Links” webpage has hyperlinks to existing online resources for Legius syndrome and *SPRED1*, including Databases, Gene and Protein Links, Web Reviews, and Literature Reviews. Some of the resources listed here include Gene Reviews, Genetics Home Reference, OMIM, UniProt, Human Protein Reference Database (HPRD), and the *SPRED1* reference sequences used for our database curation. Lastly, recent literature reviews from experts in this disease area are posted and updated as appropriate.

The *SPRED1* database can be displayed and searched in its entirety by accessing the “Database” webpage shown in Figure 1B. The database contained 78 entries as of May 2011. The default database display is by genomic position of each sequence variant (5′ to 3′) within the *SPRED1* gene. Figure 1C shows the database display columns of Location, Mutation Type, Nucleotide Change, Protein Change, Classification, Meets NIH NF1 Criteria, References, and Comments. Information in the Comments column (Figure 1D) is available by placing the pointer over the comments icon. This information may further explain the data displayed for each entry. The database also has “live search” functionality, so that any term entered in the search box (*e.g.*, *exon*, *insertion*, *benign*, or author name), will dynamically filter the displayed listing. Finally, clicking any column heading will sort (ascending and descending) the gene variant entries for redisplay.

The “Submissions” webpage is used for submission of novel *SPRED1* sequence variants to the database or to update information for sequence variations currently archived in the database. Contact information is conveniently seen on the “Home” and “Links” pages. Requested information during submission includes gene variant location, mutation type, classification, nucleotide change, protein change, clinical significance, and contact information of the submitter. All submissions are received via autogenerated e-mail to the curators, and variant updates are added to the database quarterly.

### Database display columns

#### SPRED1 sequence variation location and mutation type:

**Figure 2:**
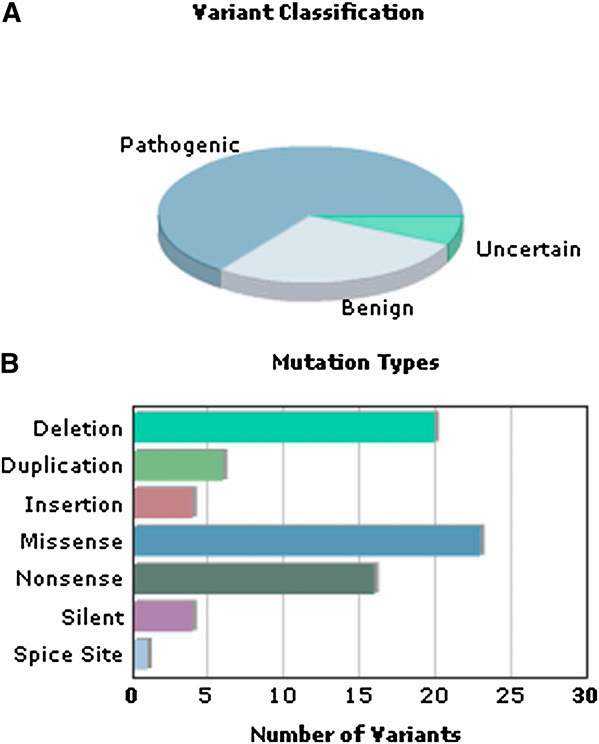
Dynamic graphical summary of (A) variant classification and (B) types of mutations found in the *SPRED1* database.

The Location column displays the exon or intron number for each *SPRED1* gene variant. Mutation Type describes the biological event leading to the change in the DNA (relative to the reference sequence). Common types of variants include small deletions and insertions/duplications, missense, nonsense, splice-site, silent, and recently, large deletions (those equal to or larger than a single exon) that have been reported (Spencer *et al.* 2011). Large duplications have not been reported in individuals with Legius syndrome. *SPRED1* gene variant mutation types are graphically summarized in Figure 2.

#### SPRED1 genotype:

The Nucleotide Change column displays the corresponding HGVS nomenclature (Den Dunnen and Antonarakis 2000). For cDNA numbering in the Nucleotide Change column of the *SPRED1* database, the first cDNA nucleotide (c.1) is the “A” of the ATG initiation (start) codon.

#### SPRED1 protein change:

For the Protein Change column, the single amino acid changes are listed in the database display. Where splice-site variants occur, only the genotype is listed, with the protein change indicated with a question mark.

#### Classification of pathogenicity:

In the Classification column, definitions for pathogenicity are Pathogenic, Suspected Pathogenic, Benign, Suspected Benign, or Uncertain. As is common practice, loss of function (LOF) mutations are assumed pathogenic, and the author’s classification for each variant was used.

#### Legius syndrome phenotype:

The phenotype associated with each variant is listed by author if the variant was reported more than once.

#### References and comments:

The final two columns of the database feature links to literature resources and genotype/phenotype findings of each individual variant. As displayed in Figure 1D, moving the cursor over the comments icon highlights a key feature and unique part of this online resource. Comments for each *SPRED1* variant often contain additional information or evidence to support the classification designation and whether the patient meets NIH diagnostic criteria for a clinical diagnosis of NF1. Importantly, clinical details, such as age or other conditions, may be included. Sensitive and private patient information is never listed.

### Database contents

Currently, the database displays information and classification of 78 *SPRED1* gene variants. However, this number is actively increasing as additional variants are described in the scientific literature and discovered in routine clinical testing and research laboratories. Pathogenic mutations detected during routine clinical testing of patient samples at ARUP Laboratories or those submitted by other laboratories will also be added to the database. As mentioned above, for variants included from the scientific literature, the database provides a linked reference to the PubMed abstract for each variant. Because the classification of each variant is directly based on evidence from the scientific literature, the link to PubMed is a very useful feature of this database. However, in cases where a variant has not yet been reported in the literature, the database provides the clinical and/or experimental, functional data suggestive of its classification. Laboratories and database users may also contact curators via e-mail or phone regarding database entries, additional references, or clarification of information posted on the site at any time.

Figure 2 summarizes the content of the *SPRED1* database by variant classification and mutation types. Analysis of the different types of mutations currently contained in the database are provided, including deletion (16), large deletion (3), insertion/deletion (1), duplication (6), insertion (4), missense (23), nonsense (16), silent (4), intronic (4) and splice site effect (1).

### Clinical significance of the database

Clinical and research laboratories that offer full gene analysis often encounter rare variants and must rely on the published literature or online resources to make informed decisions about the classification of the variant when reporting their findings. We developed this database to facilitate this decision-making process and assist those who interpret results and care for patients. A key feature of this database is the ease of access with which it provides pertinent gene variant information and the associated genotype-phenotype observations. In addition, the database is curated by individuals with expertise in molecular genetics and Legius syndrome, ensuring that all of the information contained in the archive is up to date and of sufficient scientific rigor. Finally, as this database is a publicly available resource, with no login or membership requirement, it serves as an ideal centralized resource to all laboratories offering *SPRED1* gene analysis, as well as to genetic counselors or physicians treating patients.
